# ROLE OF FAMILY CHARACTERISTICS OF RURAL AND URBAN NURSING HOME RESIDENTS AT END-OF-LIFE (EOL)

**DOI:** 10.1093/geroni/igae098.2217

**Published:** 2024-12-31

**Authors:** Caroline Stephens, Rebecca Utz, Mike Hollingshaus, Djin Tay, Linda S Edelman, Eli Iacob, Ken Smith, Katherine Ornstein

**Affiliations:** University of Utah, Salt Lake City, Utah, United States; University of Utah, Salt Lake City, Utah, United States; University of Utah, Salt Lake City, Utah, United States; University of Utah, Salt Lake City, Utah, United States; College of Nursing, Salt Lake City, Utah, United States; University of Utah, Salt Lake City, Utah, United States; University of Utah, Salt Lake City, Utah, United States; Johns Hopkins University, Baltimore, Maryland, United States

## Abstract

Nursing home (NH) EOL disparities are an increasingly important focus for policymakers. Despite the critical role of families in resident EOL care, no studies to date have explored how family size and characteristics impact EOL care utilization for urban vs rural NH residents. This study describes the size and characteristics of first-degree families (FDF) of Utah NH residents who died in rural/frontier (n=10,440) vs. urban (n=32,366) NHs between 1998-2016. Using the Utah Caregiving Population Study (Utah C-PopS), we linked NH decedents to their FDF (urban FDF n=90,829; children=57%; siblings=32%; spouses=11%; rural FDF n=30,512; children=55%; siblings=34%; spouses=11%) and compared characteristics of those with and without FDF by urban and rural status. Rates of ER visits and hospitalizations in the last six months of life by FDF type were estimated. Rural NH decedents were slightly more likely to lack FDF in the state compared to urban NH decedents (23% vs 22%, respectively). Having a spouse was associated with increased rates of hospitalization among both urban [IRR 1.12 (1.06,1.17)] and rural NH decedents [IRR 1.11 (1.03,1.19)], but increased rates of ED visits [IRR 1.10 (1.01,1.20)] were only observed among urban decedents. Having at least one child, or a child and spouse, was associated with increased rates of hospitalization in urban decedents [IRR 1.06 (1.03,1.10), IRR 1.13 (1.08,1.19), respectively], but not among rural NH decedents. A better understanding of family role in EOL care in the NH setting will advance research on disparities in EOL NH care, particularly related to rural and urban differences.

